# Association of gastroesophageal reflux disease with increased risk of chronic otitis media with effusion in adults: A nationwide population-based cohort study

**DOI:** 10.1097/MD.0000000000026727

**Published:** 2021-07-23

**Authors:** Cha Dong Yeo, Jong Seung Kim, Eun Jung Lee

**Affiliations:** a Department of Otorhinolaryngology-Head and Neck Surgery, College of Medicine, Jeonbuk National University, Jeonju, Republic of Korea; b Research Institute of Clinical Medicine of Jeonbuk National University-Biomedical Research Institute, Jeonbuk National University Hospital, Jeonju, Republic of Korea; c Department of Medical Informatics, College of Medicine, Jeonbuk National University, Jeonju, Republic of Korea.

**Keywords:** chronic otitis media with effusion, cohort, gastroesophageal reflux disease, nationwide, risk factors

## Abstract

This study aimed to evaluate the risk of developing chronic otitis media with effusion (OME) in individuals with gastroesophageal reflux disease (GERD).

A retrospective propensity score-matched cohort study was performed using data from the Korea National Health Insurance Service. The GERD group (n = 3532) included certain individuals who had been diagnosed with GERD between January 2002 and December 2005. A comparison control group (n = 14,128) was calculated by 1:4 Propensity Score (PS) matching considering age, sex, and comorbidities and year of enrolment. Each patient was monitored until 2013. Survival analysis, the log-rank test, and Cox proportional hazard regression models were used to calculate the incidence, survival rate, and hazard ratio (HR) of chronic OME for each group.

Among the 17,660 individuals included in the study population (53.2% male), the overall incidence of chronic OME during the 11-year follow-up was 1.84-fold higher in the GERD group than in the non-GERD group (1.8 vs 3.0 per 1000 person-year; adjusted HR 1.84; 95% confidence interval [CI], 1.46–2.31). Moreover, the adjusted HRs of developing chronic OME (allergic rhinitis, 1.69 [95% CI, 1.37–2.10]; asthma, 1.29 [95% CI, 1.02–1.64]; chronic rhinosinusitis, 1.61 [95% CI, 1.26–2.05]) were greater in study population with comorbidities.

From long-term follow-up, the prevalence of chronic OME in adults was 1.84 times higher in the GERD group compared with the non-GERD group. Specifically, it found that allergic rhinitis, asthma, or chronic rhinosinusitis showed increase the risk of developing chronic OME than those without these conditions.

## 1 Introduction

Gastroesophageal reflux of the stomach contents is considered to induce certain manifestations of supraesophageal lesions. These lesions can be found in the pharynx, larynx, nasal cavity, or middle ear.^[1]^ High pepsin/pepsinogen concentrations in the middle ear effusions of children were first reported in 2002 by Tasker et al,^[2]^ who proposed a contributory relationship between otitis media with effusion (OME) and gastroesophageal reflux disease (GERD) in children.

Compared with the many studies of OME and its relationship with reflux disease in children, studies in adults are very scarce. In 2001, Poelmans and colleagues^[3]^ were the first to report adult patients with middle ear disease which was suspected to have been caused by GERD. Patients with GERD and chronic ear disease were identified by endoscopic examination and oesophageal pH monitoring over a 24-hour period. All four patients responded well to anti-GERD treatment.

Many studies have reported that GERD affects OME in children, but there has been no study on the correlation between GERD in adults and chronic OME using large-scale real-world data. Our study started with the assumption that GERD in adults and chronic OME may be related.

The objective of this study was to evaluate the risk of chronic OME in GERD and to identify which comorbidities increased the risk of chronic OME. This study is expected to aid our understanding of the relationship between GERD and chronic OME.

## 2 Methods

### 2.1 Korea national health insurance service

Korea national health insurance service (KNHIS) is Korea's health insurance service, established by the Korean government in 1963, and since 1989, almost everyone in Korea has been enrolled. This database includes information such as the identification number of the individual, sex, age, residential area, and income quintiles, as well as diagnostic code, treatment history, prescription details including medication, and cost. KNHIS collects data by anonymising it using a person-id to replace the 13-digit identification number of each individual. In KNHIS, the Korean Standard Classification of Disease (KCD) is used as the diagnostic code, similar to the WHO International Classification of Diseases, 10th revision (ICD-10). The National Health Insurance Service-National Sample Cohort (NHIS-NSC) consists of about 1 million people, 2% of the total 50 million people in Korea, whose data on age, sex, residential area, and economic status were extracted randomly from the database. This retrospective cohort study contains basic demographic variables such as the patient's age, sex, complex information such as the patient's visit date, diagnosis code, treatment history, medications, and insurance claims.

### 2.2 Study population

The definition of GERD, chronic OME and comorbidities (allergic rhinitis, asthma, and chronic rhinosinusitis) used the diagnostic code (based on the Korean Standard Classification of Disease [KCD] diagnosis code) in NHIS-NSC insurance claim data.

The GERD group included all patients who received inpatient or outpatient care for an initial diagnosis of GERD between January 1, 2002 and December 31, 2005. To improve the accuracy of the diagnosis, a positive GERD diagnosis satisfied all of the following conditions:

1. the patient received a diagnosis under KCD code K21, K210, or K219;
2. the patient underwent at least one of the following tests (esophageal 24-hour pH monitoring, upper gastrointestinal endoscopy, and laryngoscopy); and
3. the patient was prescribed H2 receptor blocker or proton pump inhibitor (PPI) medication over 28 days.

Patients were excluded under the following criteria:

1. younger than 20 years;
2. a diagnosis of chronic OME (KCD codes H652, H653) between January 1, 2002 and December 31, 2005, before the diagnosis of GERD;
3. death from any cause between January 1, 2002 and December 31, 2005.

From the GERD cohort defined above, the control group was calculated by 1:4 Propensity Score (PS) matching considering age, sex, and comorbidities. We performed PS matching using a “greedy nearest neighbour” algorithm with a 1:4 ratio. Finally, 3532 eligible patients with GERD and 14,128 patients in the comparison group were enrolled. Each patient was tracked until December 31, 2013, or until the occurrence of chronic OME was recorded. In addition, we included only patients who were diagnosed with chronic OME by a paediatrician or by an otolaryngologist.

### 2.3 Predictor and outcome variables

Details of patients’ age, sex, and comorbidities were obtained from the database. The study population was divided into 6 age groups (20–29, 30–39, 40–49, 50–59, 60–69, and ≥70 years). We analyzed comorbidities, including allergic rhinitis (KCD code J30), asthma (KCD J45, J46), chronic rhinosinusitis (CRS) (KCD code J32 or J33), and adenotonsillitis (KCD code J35), which are all known risk factors for chronic OME. We defined the presence of comorbidities as any diagnoses of these codes between January 1, 2002 and December 31, 2005, before the diagnosis of chronic OME.

The working definition of the study end point was a diagnosis of chronic OME by any cause. Patients were excluded after December 31, 2013 if they did not endure any events and if they were still alive at that date. The risks of chronic OME in the GERD and control groups were compared as person-years at risk, which were defined as the period between either the date of GERD diagnosis or the date of first hospital visit (for the comparison group), and the study end point.

### 2.4 Statistical analysis

Kaplan–Meier analysis was performed to identify the difference in survival functions among the study groups. The Kaplan–Meier failure curve for the observation period and the log-rank test were used to assess the difference between curves. To determine whether GERD increased the risk of occurrence of chronic OME, we calculated the hazard ratio (HR) and 95% confidence interval, adjusted for the other predictor variables, using Cox proportional hazard regression analyses. Incidence rates per 1000 person-years for chronic OME were obtained by dividing the number of patients with chronic OME by person-years at risk. Data management and statistical analyses were performed in STATA (version 16.0; StataCorp, College Station, TX).

### 2.5 Ethical considerations

All studies were conducted and designed in accordance with the Declaration of Helsinki using KNHIS-NSC data. The research was also approved by the Institutional Review Board of Jeonbuk National University Hospital (IRB file number 2019-04-056). Informed consent was waived by the Institutional Review Board which approved the study.

## 3 Results

**Figure 1:**
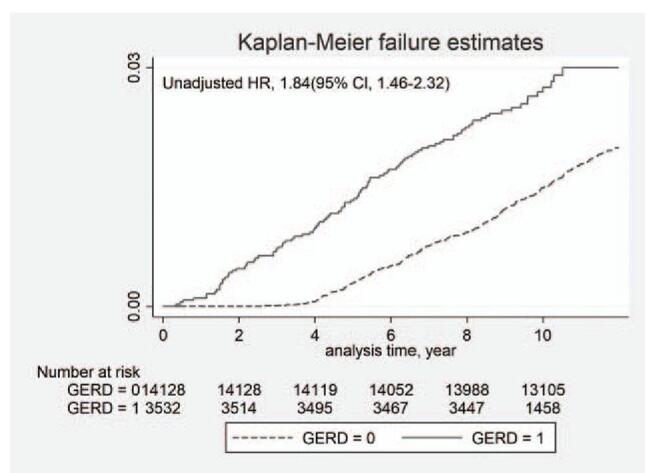
Kaplan–Meier failure curves of patients in GERD and non-GERD groups (GERD group = 1, non-GERD group = 0); GERD = gastroesophageal reflux disease.

The present study consisted of 3532 patients with GERD and 14,128 in the control comparison group for a total study population of 17,660 (53.2% male and 46.8% female). The two groups had similar distributions of sex, age, and comorbidities. Details of the study population and group characteristics are presented in Table 1. Kaplan–Meier failure curves with log-rank tests for the 11-year follow-up period are presented in Figure 1 (unadjusted HR, 1.84; 95% CI 1.46–2.32). The log-rank test indicated that the patients with GERD developed chronic OME more frequently than the control group (*P* < .05).

**Table 1:** Characteristics of study patients.

	Comparison Group (n = 14128)		GERD Group (n = 3532)			
Variable	n	%	n	%	Chi squared	*P*
Sex						
Male	7517	53.2	1880	53.2	0.0005	.982
Female	6611	46.8	1652	46.8		
Age, years						
20-29	1444	10.2	361	10.2	0.0080	1.000
30-39	2748	19.5	687	19.5		
40-49	3888	27.5	972	27.5		
50-59	3188	22.6	797	22.6		
60-69	2380	16.8	596	16.9		
≥70	480	3.4	119	3.4		
Comorbidities						
Allergic rhinitis						
No	7936	56.2	1984	56.2	0.0000	1.000
Yes	6192	43.8	1548	43.8		
Asthma						
No	11834	83.8	2959	83.8	0.0004	.984
Yes	2294	16.2	573	16.2		
Adenotonsillitis						
No	13592	96.2	3398	96.2	0.0000	1.000
Yes	536	3.8	134	3.8		
Chronic rhinosinusitis						
No	12199	86.3	3049	86.3	0.0011	.974
Yes	1929	13.7	483	13.7		
Total	14128		3532			

GERD = gastroesophageal reflux disease.

The HR was analyzed for development of chronic OME during the 11-year follow-up period using univariate and multivariate Cox regression models and these findings are presented in Table 2. The overall incidence of chronic OME was significantly higher in the GERD group (3.0 per 1000 person-years) than in the control group (1.8 per 1000 person-years), with an unadjusted HR of 1.84 (95% CI, 1.46–2.32). After adjustment for sociodemographic factors (sex and age) and comorbidities, the GERD group showed a significant association with the prospective development of chronic OME (HR, 1.84; 95% CI, 1.46–2.31). We also found that increasing age was significantly associated with the prospective development of chronic OME (50–59 years: HR, 1.63; 95% CI, 1.09–2.45; and 60–69 years: HR, 1.55; 95% CI, 1.02–2.36). Patient comorbidities were also significantly related to the prospective development of chronic OME (allergic rhinitis: HR, 1.69; 95% CI, 1.37–2.10; asthma: HR, 1.29; 95% CI, 1.02–1.64; and chronic rhinosinusitis: HR, 1.61; 95% CI, 1.26–2.05).

**Table 2:** Incidence per 1000 person-years and hazard ratios (HR) of chronic otitis media with effusion during 11-year follow-up period.

				HR (95%CI)	
	No. of participants	No. of cases	Incidence, per 1000 person-years	Unadjusted	Adjusted
Comparison group	14,128	284	1.8	1 [Reference]	1 [Reference]
GERD group	3,532	101	3.0	1.84 (1.46–2.32)	1.84 (1.46–2.31)
Sex					
Male	9,397	192	1.9	1[Reference]	1[Reference]
Female	8,263	193	2.1	1.12 (0.92–1.37)	1.00 (0.82–1.23)
Age					
20–29	1,805	30	1.5	1 [Reference]	1 [Reference]
30–39	3,435	60	1.6	1.04 (0.67–1.61)	0.99 (0.64–1.54)
40–49	4,860	89	1.7	1.09 (0.72–1.64)	1.07 (0.70–1.61)
50–59	3,985	110	2.5	1.64 (1.09–2.45)	1.63 (1.09–2.45)
60–69	2,976	81	2.4	1.60 (1.05–2.43)	1.55 (1.02–2.36)
≥70	599	15	2.2	1.47 (0.79–2.73)	1.43 (0.77–2.67)
Comorbidities					
Allergic rhinitis					
No	9,920	153	1.4	1 [Reference]	1 [Reference]
Yes	7,740	232	2.7	1.90 (1.55–2.33)	1.69 (1.37–2.10)
Asthma					
No	14,793	292	1.8	1 [Reference]	1 [Reference]
Yes	2,867	93	2.9	1.61 (1.28–2.04)	1.29 (1.02–1.64)
Adenotonsillitis					
No	16,990	360	1.9	1 [Reference]	1 [Reference]
Yes	670	25	3.4	1.75 (1.17–2.62)	1.47 (0.98–2.22)
Chronic rhinosinusitis					
No	15,248	291	1.7	1 [Reference]	1 [Reference]
Yes	2,412	94	3.5	2.01 (1.60–2.54)	1.61 (1.26–2.05)

GERD = gastroesophageal reflux disease.

## 4 Discussion

Gastric cells release pepsinogen (PG) and, in the presence of gastric acid, this is converted to pepsin. When activated by acid, pepsin can directly damage supraesophageal lesions. When gastric material flows back into the nasopharynx, this can cause an inflammatory reaction, and possibly also secondary infection, resulting in dysfunction of the Eustachian tube as well as chronic otitis media.^[4]^ A study of PG concentrations in middle ear effusions (MEEs) in adult patients having chronic OME with no obvious cause indicated that PG levels were significantly higher in individuals who had gastroesophageal reflux-related symptoms than in those not having any symptoms.^[5]^ The likely mechanisms explaining the occurrence of PG in MEEs may be serum conversion or reflux. Levels of PG in the middle ear are considerably higher than concentrations in serum, indicating that serum conversion is an improbable mechanism.^[5–7]^

In an investigation using the Mongolian gerbil model, relaxation of the lower oesophageal sphincter caused reflux of gastric content and this was reported to reach the middle ear on both sides.^[8]^ Bilateral OME was shown to be present in a significantly higher proportion of patients who had gastroesophageal reflux-related symptoms than in those patients without symptoms. If acid reflux was a contributory factor in OME, bilateral OME would be a rational consequence.^[5]^

During the first acid reflux event, refluxed material in the middle ear cavity would have an acidic pH, and the Eustachian tube and middle ear mucosa would suffer transient damage leading to inflammation.^[1]^ In rats, nasopharyngeal exposure to a combination of HCl and pepsin affected the mucociliary clearance and ventilatory function of the Eustachian tube.^[9]^

The idea of gastric acid contents insulting the Eustachian tube or middle ear mucosa seems to help explain the pathological occurrence of OME. Although many studies have been conducted in children, studies in adults are still scarce. The studies in children investigated three aspects (the prevalence of GERD in children with chronic OME evaluated through pH monitoring; the prevalence of pepsin/pepsinogen in the middle ear of children with chronic OME undergoing ventilation tube insertion; and therapeutic tests with anti-GERD drugs in children with chronic OME).^[1]^

Unlike gastric acid reflux, bile reflux of duodenal contents via the stomach into the esophagus probably only accounts for 10% to 15% of non-acid reflux.^[10]^ Perfusion studies show that conjugated bile acids, in an acidic environment, produce esophageal mucosal injury, whereas unconjugated bile acids and trypsin are harmful at more neutral pH values (pH 5–8). This contribution of unconjugated bile acids and trypsin to significant esophageal mucosal injury in patients with GERD is likely minimal, because trypsin is not active and unconjugated bile acids precipitate at acidic pH values.^[11]^ Conjugated bile acids enter the mucosal cells in the unionized form (predominate from at low pH) through the lipophilic lipid membrane and then accumulate as intracellular ionization results in entrapment.^[12]^ These high concentrations of bile acids cause intracellular damage by the dissolution of cell membranes and tight junction. On the other hand, acid and activated pepsin cause deeper and more severe injury though proteolytic actions.^[13]^

Compared with the many studies of OME and its relationship with reflux disease in children, studies in adults are very scarce. In 2001, Poelmans and colleagues^[3]^ were the first to report adult patients with middle ear disease which was suspected to have been caused by GERD. Patients with GERD and chronic ear disease were identified by endoscopic examination and oesophageal pH monitoring over a 24-hour period. All four patients responded well to anti-GERD treatment. Sone et al^[14]^ documented a significant association between gastroesophageal reflux (GER) symptoms and OME of unknown etiology. Study reported relationship between body mass index (BMI) and GER-related OME, especially in elderly patients. Similar to our study, Karyanta et al^[15]^ reported that the prevalence ratio of OME in GERD group is 4.5 times that in non-GERD group. Our research is a large-scale study of adults who have been diagnosed with GERD through aforementioned examination using real-world data.

Kreiner-Moller et al^[16]^ documented a significant association between allergic rhinitis and OME; the presence of allergic rhinitis increased the risk of OME by an odds ratio >3. The association between allergic rhinitis and OME may represent a localized allergic inflammation in the respiratory epithelium of the middle ear, a secondary inflammation as a result of Eustachian tube dysfunction, or other unknown mechanisms.

Tomioka et al^[17]^ reported cases of intractable OME associated with bronchial asthma. Middle ear effusion and otorrhea in those cases contained numerous eosinophils and were very viscous. They named this condition eosinophilic otitis media. Iino et al^[18]^ found a high odds ratio of OME in bronchial asthma patients and proposed diagnostic criteria for eosinophilic otitis media. A patient who shows OME or chronic otitis media with eosinophil-dominant effusion can be diagnosed as having eosinophilic otitis media.

The incidence of OME in patients with chronic rhinosinusitis differs considerably in various studies, so the role of chronic rhinosinusitis in OME development is unclear.^[19,20]^ However, Hong et al^[21]^ reported that the rate of concomitant chronic rhinosinusitis and OME was 15.4%. They also found that adenoids, IgA, BCL-6, and squamous metaplasia are important for the development of OME.

There is a lack of research on the correlation between adenotonsillitis and OME. Marseglia et al have suggested that adenoiditis is a significant risk factor for OME development and that the risk becomes higher when allergic rhinitis and adenoiditis are concomitantly present.^[22]^ They suggested that allergic rhinitis and adenoiditis could increase the risk of OME by nasal obstruction. However, our study found that there was no significant correlation between adenotonsillitis and chronic OME.

This is the first study to use large-scale real-world data to evaluate the risk of chronic OME in GERD. Although we demonstrated significant findings, there are several limitations that should be considered in future research. First, GERD and chronic OME were identified only by the diagnostic code without information such as severity of reflux, reflux symptom index, reflux finding score or physical examination findings.^[23]^ However, we tried to improve the diagnostic accuracy of GERD by including patients who underwent at least one of the following tests: esophageal 24-hour pH monitoring, upper gastrointestinal endoscopy, and laryngoscopy, and who were prescribed anti-GERD medication for more than 4 weeks. Second, acute OME and chronic OME were identified only by the diagnostic code. But, in this study, we used the diagnostic code only assigned by the paediatrician or otolaryngologist to improve the diagnostic accuracy of OME. Third, the results of this study did not consider function of Eustachian tube, family history, smoking history, drinking history, BMI or other health-related indicators. Further research combining this information should be undertaken and would provide definitive results with regard to the effect of GERD on chronic OME.

## 5 Conclusion

This observational study indicated that GERD is associated with an increased incidence of chronic OME in adults. Specifically, we found that patients with allergic rhinitis, asthma or chronic rhinosinusitis showed a higher risk of developing chronic OME than those without these conditions.

When a patient is diagnosed as having chronic OME, doctors should bear in mind that the cause may be GERD.

## Author contributions

**Conceptualization:** Cha Dong Yeo, Jong Seung Kim, Eun Jung Lee.

**Data curation:** Cha Dong Yeo, Jong Seung Kim, Eun Jung Lee.

**Formal analysis:** Cha Dong Yeo, Jong Seung Kim, Eun Jung Lee.

**Investigation:** Cha Dong Yeo, Eun Jung Lee.

**Methodology:** Jong Seung Kim, Eun Jung Lee.

**Project administration:** Eun Jung Lee.

**Resources:** Cha Dong Yeo, Jong Seung Kim.

**Software:** Cha Dong Yeo, Jong Seung Kim.

**Supervision:** Jong Seung Kim, Eun Jung Lee.

**Validation:** Cha Dong Yeo, Jong Seung Kim, Eun Jung Lee.

**Visualization:** Cha Dong Yeo, Jong Seung Kim.

**Writing – original draft:** Cha Dong Yeo.

**Writing – review & editing:** Cha Dong Yeo, Jong Seung Kim, Eun Jung Lee.
